# Cervical cancer screening in incarcerated women: deprived of freedom or deprived of health?

**DOI:** 10.1590/0034-7167-2025-0011

**Published:** 2026-07-24

**Authors:** Elaine Regina Prudencio Hipólito da Silva, Vanessa Terezinha Gubert, Marco Antonio Moreira Puga, Ines Aparecida Tozetti

**Affiliations:** I Universidade Federal de Mato Grosso do Sul. Campo Grande, Mato Grosso do Sul, Brazil

**Keywords:** Papanicolaou Test, Squamous Intraepithelial Lesions of the Cervix, Human Papillomavirus Viruses, Prisons, Vaccines., Teste de Papanicolaou, Lesões Intraepiteliais Escamosas Cervicais, Papilomavírus Humanos, Prisões, Vacinas., Prueba de Papanicolaou, Lesiones Intraepiteliales Escamosas de Cuello Uterino, Virus del Papiloma Humano, Prisiones, Vacunas.

## Abstract

**Objectives:**

to reflect on and discuss the reality of cervical cancer screening among incarcerated women.

**Methods:**

theoretical-reflective study, developed from a previous review of the scientific and documentary literature on cervical cancer screening programs, encompassing international, Latin American, and Brazilian experiences. The analysis and reflection were conducted critically, aiming to identify advances, limitations, and gaps in screening and treatment strategies.

**Results:**

although the prison environment facilitates the development of cervical cancer screening actions, as it contributes to access to women, they have a lower chance of being investigated, indicating a possible irregularity in the supply, suggesting a weakness and inconsistency in healthcare. The purpose of incarceration may be to deprive offenders of freedom, but not to deny their rights to health.

**Final Considerations:**

the reflections presented here emphasize the importance of nursing in conducting screening, follow-up, and health education actions, particularly among populations in situations of greater vulnerability.

## RESUMO

**Objetivos:**

refletir e discutir a realidade do rastreamento do câncer do colo do útero em mulheres privadas de liberdade.

**Métodos:**

estudo teórico-reflexivo, desenvolvido por revisão prévia da literatura científica e documental sobre rastreamento do câncer do colo do útero, abrangendo experiências internacionais, latino-americanas e brasileiras. A análise e a reflexão foram conduzidas de forma crítica, para identificar avanços, limitações e lacunas nas estratégias de rastreamento e tratamento.

**Resultados:**

embora o ambiente prisional facilite o desenvolvimento de ações de rastreamento, por favorecer o acesso às mulheres, elas apresentam menor probabilidade de serem investigadas, indicando possível irregularidade na oferta, fragilidade e inconsistência no cuidado em saúde. A finalidade do encarceramento pode ser a privação de liberdade, mas não a negação dos direitos à saúde.

**Considerações Finais:**

as reflexões apresentadas destacam a importância da enfermagem na realização de ações de rastreamento, acompanhamento e educação em saúde, especialmente entre populações de maior vulnerabilidade.

## RESUMEN

**Objetivos:**

reflexionar sobre la realidad de la detección del cáncer cervicouterino en mujeres privadas de libertad.

**Métodos:**

estudio teórico-reflexivo basado en una revisión de la literatura científica y documental sobre programas de detección de cáncer cervicouterino a nivel internacional, latinoamericano y brasileño. El análisis y la reflexión críticos identificaron avances, limitaciones y deficiencias en las estrategias de detección y tratamiento.

**Resultados:**

aunque el entorno penitenciario facilita el acceso a servicios de detección del cáncer cervicouterino, la baja tasa de examinación indica una irregularidad en la oferta y fragilidad e inconsistencia en la atención médica. Si bien el encarcelamiento implica la privación de libertad, esto no exime de garantizar el derecho a la salud.

**Consideraciones Finales:**

las reflexiones enfatizan la importancia de la enfermería en la realización de acciones de detección, seguimiento y educación para la salud, particularmente entre poblaciones en situaciones de mayor vulnerabilidad.

## INTRODUCTION

Globally, cervical cancer is the fourth-most-common type of cancer and the fourth-leading cause of death among women, with an estimated 604 000 new cases and 342 000 related deaths in 2020. Persistent Human papillomavirus (HPV) infection is a major risk factor for cervical dysplasia and carcinogenesis^(1)^.

Early diagnosis increases the likelihood of cure, as the course of cervical cancer is characterized by slow progression and well-defined phases that facilitate timely identification of cellular changes. Vaccination is also a valuable tool in HPV prevention, not only by decreasing the number of infected individuals, but also by reducing the burden of costs of diagnosis and treatment of cervical diseases^(1)^.

In addition to experiencing confinement-related constraints, incarcerated women face inadequate access to healthcare. Prevalence of HPV in this population varies by country, with infection rates of roughly 10.5% in Brazil and 55.4% in Taiwan. In the general population, the global prevalence of HPV infection among women with normal cytology is 11.4%, suggesting that rates among incarcerated women are high^(2)^.

It is equally crucial to devise strategies that ensure incarcerated women have adequate access to these services.

## OBJECTIVES

To reflect on and discuss the reality of cervical cancer screening among incarcerated women.

## METHODS

This is a theoretical-reflective study, was developed from a previous review of the scientific and documentary literature on cervical cancer screening programs, encompassing international, Latin American, and Brazilian experiences. The analysis and reflection were conducted critically, aiming to identify advances, limitations, and gaps in screening and treatment strategies, with emphasis on their implications for health services organization and for prevention among vulnerable populations, such as incarcerated women.

To support the development of this reflection, scientific articles indexed in the National Library of Medicine (PubMed) database were selected, with the aim of identifying relevant publications on cervical cancer and its screening in prison settings. An advanced search was conducted using the following controlled descriptors in English: “cervical cancer” and “screening” and “prison”.

The article selection was based on the following inclusion criteria: articles whose titles and/or abstracts addressed cervical cancer, as well as screening and prevention programs within prisons.

The screening of the retrieved articles was carried out through the reading of titles and abstracts, according to the established inclusion criteria. Subsequently, a full-text reading of the selected articles was conducted, followed by the organization and structuring of a spreadsheet summarizing the main aspects discussed in the studies, in order to enable a more effective descriptive and critical analysis.

## RESULTS

### Cervical cancer

As expected, social inequalities, including disparities in access to healthcare, have a detrimental impact on women’s health. In any given country, the incidence of cervical cancer is shaped both by women’s exposure to risk factors and by the effectiveness of local screening programs. The disease is preceded by an extensive pre-invasive period characterized by cervical intraepithelial neoplasia, also termed squamous intraepithelial lesions (SIL)^(1,2)^.

The success of cervical cancer screening and treatment programs hinges on high coverage of the female population, efficient follow-up of women with abnormal test results, effective coordination among the actors involved, and commensurate resources^(1)^.

Screening is a valuable tool for identifying treatable abnormalities and detecting precursor lesions and early-stage invasive cervical cancer, in addition to significantly reducing the incidence, morbidity, and mortality of the disease. These goals, however, are intrinsically linked with effective organization of services and equity of access to healthcare^(1)^.

Screening programs for cervical cancer were first introduced in the USA and Europe, in the 1950s. In the decade that followed, the Pan-American Health Organization recognized this cancer type as a public health problem in Latin American countries and the need for implementation of control measures^(3)^.

Incarcerated women are four times more likely to develop cervical cancer than the general female population^(4)^. In the United States, a rate of abnormal cytology for any abnormality was 35.6% and high-grade squamous intraepithelial lesion (HSIL) was 10.0% in incarcerated women, while in the control group these rates were 18.5% and 2.9%^(1)^. In the Northern Region of Brazil, the prevalence of low-grade squamous intraepithelial lesion (LSIL) and HSIL was 17.5% and 1%, respectively in incarcerated women^(5)^.

These epidemiological disparities cannot be interpreted solely through a biomedical lens. The overrepresentation of cervical lesions among incarcerated women reflects not only biological vulnerability but also the cumulative effects of structural violence, gender inequality, and selective criminalization. In Brazil, the female prison population is predominantly composed of young, Black, low-income women with limited access to education and healthcare prior to incarceration. This profile is not incidental - it is result of a punitive logic that disproportionally targets marginalized groups while failing to guarantee their fundamental rights. Thus, the high prevalence of cervical abnormalities in this population must be understood as a manifestation of broader social injustices that precede and persist throughout incarceration.

In Brazil, a countrywide strategy to address cervical cancer was implemented by the Ministry of Health only in 1972-1976 under the National Program for Cervical Cancer Control, which centered on Pap-based screening in women aged 25 to 64 years who have begun sexual activity. After two consecutive negative annual tests, subsequent exams can be performed at three-year intervals^(2)^.

Cytology-based screening programs, including Pap tests alone, have successfully helped reduce mortality in high-income countries in recent decades. By contrast, cervical cancer control in lowand middle-income countries has been hampered by underfunding and insufficient infrastructure to implement screening programs^(6)^.

Although incidence levels have remained high in lowand middle-income countries, the timely use of cytological screening methods has promoted a considerable decrease in rates. The risk of progression to cancer in a woman with cervical lesions can be reduced by 46% with a single examination in her lifetime^(6)^.

Studies have suggested that incarcerated women can benefit from a Pap test on admission and annually thereafter, given the high prevalence of abnormal cytological findings and risk factors for cervical cancer development in this group compared with the female population at large^(2)^.

Regarding cervical cancer screening, a study in women’s prisons in the Central-West region of Brazil found that only half of the participants underwent the Pap test in the prison environment. Limited opportunity was cited as the main reason for non-completion^(1)^. The absence of effective public policies aimed at the health of incarcerated women reveals not only an operational failure, but also a structural neglect that perpetuates the social and healthcare exclusion of this population.

### HPV vaccination

Vaccination for HPV prevention was approved in the United States in 2006 and in Australia in 2007. Brazil had to wait until 2014 for the quadrivalent vaccine to be included in the National Immunization Program, and thus available free of charge under the Brazilian Unified Healthcare System (SUS)^(7)^.

In alignment with the World Health Organization’s (WHO) global strategy to eliminate cervical cancer by 2030, the National Immunization Program (PIN) in mid-2024 adopted a single-dose HPV vaccination schedule for all children and adolescents aged 9 to 14 years. As part of a temporary catch-up strategy, the program was also extended to include adolescents aged 15 to 19 years who had not previously received the vaccine^(8)^.

Additionally, the PIN provides HPV vaccination to specific high-risk groups, including: individuals aged 2 years and older diagnosed with recurrent respiratory papillomatosis (RRP); individuals aged 15 to 45 years using pre-exposure prophylaxis (PrEP); victims of sexual violence aged 9 to 45 years; people living with HIV/AIDS; cancer patients undergoing chemotherapy and/or radiotherapy; and recipients of bone marrow or solid organ transplants^(7)^.

The National Immunization Program aims to achieve HPV vaccination coverage of at least 80% for the first and second doses. In 2014, this goal was reached in 87% of Brazil’s counties for the first dose, but in only 32% for the second.

Studies on HPV vaccination in prison environments are still scarce and incipient. A survey conducted at an American prison revealed that 41% of men and 70% of women had information on the vaccine, but only 13% of all inmates reported being advised by a medical professional to be vaccinated^(9)^.

In Brazil, government-funded programs for HPV vaccination target children and adolescents aged 9 to 14 years, an age bracket that renders incarcerated women ineligible as candidates for this preventive measure. This scenario underscores the need to broaden cervical cancer screening strategies, given the epidemiological profile of the disease and the current inequities in the access of these women to healthcare services.

### Incarcerated women

Worldwide, over 10 million people are currently incarcerated, and Brazil ranks third in the total number of inmates (815.165). Although males comprise the overwhelming majority (94.45%) of the Brazilian prison population, the country has the fourth-largest female incarcerated population in the world, at just over 45 000^(2)^.

Social traits (including low educational level, poor access to healthcare, scant knowledge of sexually transmitted infections) and behavioral profile (*e.g.*, early sexual debut, unprotected intercourse, high number of sexual partners, smoking, alcohol consumption) can increase women’s vulnerability to HPV, and consequently to cervical cancer^.^ These unfavorable features are typical among incarcerated women, placing them at particularly higher risk. Although closed environments, such as prisons, would presumably facilitate the implementation of screening interventions, women inmates are actually less likely to be examined for cervical cancer^(2,7)^.

The persistent underdiagnoses and limited access to preventive care among incarcerated women cannot be fully understood without considering the symbolic dimension of their exclusion. The social stigma historically associated with female incarceration - often rooted in moralistic and gender-biased narratives - contributes to their invisibility in public health policies. This stigmatization reinforces the perception that these women are less deserving of care, legitimizing institutional omissions and weakening the implementation of equity-based health strategies. Addressing this issue requires not only structural reforms but also a shift in the sociocultural paradigms that shape health policy and practice.

Although the National Policy for Comprehensive Healthcare for Persons Deprived of Liberty (PNAISP) and the SUS establish the right to comprehensive and equitable healthcare, the reality observed in the prison system reveals a persistent gap between policy and practice. This disconnection is particularly evident in the field of preventive women’s health, where the lack of structured and continuous screening programs for cervical cancer and limited access to HPV vaccination reflect institutional weakness. The failure to operationalize these policies in prison settings not only compromises the effectiveness of public health strategies but also reinforces historical patterns of exclusion and neglect toward incarcerated women. This scenario underscores the need for articulation and accountability mechanisms to ensure that the principles of universality, equity, and integrality-pillars of SUS-are effectively upheld within the prison context.

In the United States, the Federal Bureau of Prisons recommends screening upon admission, with further Pap tests recommended every three years for women aged 21-65 years. In the 30-65 age group, this interval can be extended to five years if both Pap and molecular testing for HPV are available. For HIV-positive women, annual screening is recommended regardless of age^(5)^.

In Northern Brazil, prevalence rates of 17.5% for low-grade squamous intraepithelial lesion (LSIL) and 1% for high-grade squamous intraepithelial lesion (HSIL) have been observed, whereas rates of 0.8% for LSIL and 0.26% for HSIL have been found among the general female population^(2)^.

In Midwest Brazil, only 50% of women had undergone a Pap test while incarcerated. Not having the opportunity was cited as the main reason for women not taking the testing. The longer the time served in prison, the greater the chances of a woman being tested, a pattern that suggests irregular supply as a result of shortcomings in healthcare delivery^(7)^.

In Brazil, the unit cost of a conventional cytology test for screening was roughly US$13.16 in early 2022, while treatment of invasive cervical cancer was estimated at US$2,219.73 a year-a stark statistic in a country where screening strategies have been only partially implemented and where financial resources are limited^(10)^.

Although the prison environment facilitates the development of cervical cancer screening actions, as it contributes to access to women, they have a lower chance of being investigated^(4)^, indicating a possible irregularity in the supply, suggesting a weakness and inconsistency in healthcare^(7)^. Also, the lack of systematic provision of preventive screenings and immunizations constitutes a violation of the principle of distributive justice, as it denies incarcerated women equitable access to essential health services, reinforcing structural barriers and perpetuating health inequities within the prison system.

The purpose of incarceration may be to deprive offenders of freedom, but not to deny their rights to health. The female prison population should have their intrinsic right to healthcare access respected and be able to enjoy the same level of care available to other citizens. This includes access to cervical cancer control measures.

One of the study’s limitations is the limited number of scientific publications, both in Brazil and internationally, addressing cervical cancer screening and HPV vaccination among incarcerated women. Although the study is based on a theoretical-reflective approach grounded in previous research, this limitation may constrain the depth and scope of reflections regarding interventions targeting this population across different prison contexts.

## FINAL CONSIDERATIONS

Incarcerated women remain vulnerable to developing cervical cancer, a preventable disease that can be screened at a relatively low cost and has a high potential for cure. In this context, it is essential to align national strategies with the World Health Organization’s Global Strategy to Accelerate the Elimination of Cervical Cancer as Public Health Problem, with sets ambitious targets to be achieved by 2030, including 90% HPV vaccine coverage, 70% screening with a high-performance tests, and 90% treatment for identified cases^(8)^.

The exclusion of incarcerated women from these preventive measures not only undermines the achievement of these goals but also perpetuates cycles of health inequity. Integrating prison health into national cancer control plans is therefore a necessary step toward universal health coverage and the ethical imperative of leaving no one behind. Well-designed strategies for screening and prevention in prison environments can swiftly improve access to diagnosis and timely treatment.

There is a pressing need for training of prison healthcare teams on collecting samples, interpreting test results, and conducting interventions, given the central role of screening in detecting cervical cancer and precursor lesions.

Although most women incarcerated in Brazil are not currently eligible for HPV vaccination, information on their immunization status before admission might help widen the coverage of cervical cancer screening.

Identifying and immunizing women inmates affected by other conditions, including HIV carriers, those undergoing cancer treatment, and immunosuppressed women, should help reduce the prevalence of HPV in this population.

Implementing measures to improve the knowledge on cervical cancer and its risk factors held by this neglected population, besides raising awareness on the importance of screening and vaccination, are just some of the actions required to empower these women to embrace self-care in their lives and demand that their fundamental rights to health be met.

The reflections presented here emphasize the importance of nursing in conducting screening, follow-up, and health education actions, particularly among populations in situations of greater vulnerability. Evidence-based professional practice, combined with the ethical and social commitment of nursing, serves as a strategic axis for expanding access, improving the quality of care, and enhancing the effectiveness of public policies aimed at the prevention of cervical cancer.

## Data Availability

The research data are available only upon request.

## References

[1] Bedell SL, Goldstein LS, Goldstein AR, Goldstein AT. (2020). Cervical cancer screening: past, present, and future. Sex Med Rev.

[2] Escobar N, Plugge E. (2020). Prevalence of human papillomavirus infection, cervical intraepithelial neoplasia and cervical cancer in imprisoned women worldwide: a systematic review and meta-analysis. J Epidemiol Community Health.

[3] Fair H, Walmsley R. (2021). World prison population list.

[4] Federal Bureau of Prisons (2018). BOP Preventive Health Care Screening: clinical guidance.

[5] Fontham ETH, Wolf AMD, Church TR, Church TR, Etzioni R, Flowers CR (2020). Cervical cancer screening for individuals at average risk: 2020 guideline update from the American Cancer Society. CA Cancer J Clin.

[6] Jodry D, Blemur D, Nguyen ML, Kuhn T, Easley K, Wang H (2021). Criminal justice involvement and abnormal cervical cancer screening results among women in an urban safety net hospital. J Low Genit Tract Dis.

[7] Moura LL, Codeço CT, Luz PM. (2021). Cobertura da vacina contra papilomavírus humano (HPV) no Brasil: heterogeneidade espacial e entre coortes etárias. Rev Bras Epidemiol.

[8] Levi M. Esclarecimento sobre esquemas das vacinas HPV em vigor. Sociedade Brasileira de Imunizações; 2024.

[9] Sung H, Ferlay J, Siegel RL, Laversanne M, Soerjomataram I, Jemal A (2021). Global cancer statistics 2020: GLOBOCAN Estimates of incidence and mortality worldwide for 36 cancers in 185 countries. CA Cancer J Clin.

[10] Wilailak S, Kengsakul M, Kehoe S. (2021). Worldwide initiatives to eliminate cervical cancer. Int J Gynaecol Obstet.

